# Antagonistic Effects of *Corynebacterium pseudodiphtheriticum* 090104 on Respiratory Pathogens

**DOI:** 10.3390/microorganisms12071295

**Published:** 2024-06-26

**Authors:** Ramiro Ortiz Moyano, Stefania Dentice Maidana, Yoshiya Imamura, Mariano Elean, Fu Namai, Yoshihito Suda, Keita Nishiyama, Vyacheslav Melnikov, Haruki Kitazawa, Julio Villena

**Affiliations:** 1 Laboratory of Immunobiotechnology, Reference Centre for Lactobacilli (CERELA-CONICET), San Miguel de Tucumán 4000, Argentina; rortiz@cerela.org.ar (R.O.M.); stefi.dentice@gmail.com (S.D.M.); melean@cerela.org.ar (M.E.); 2 Food and Feed Immunology Group, Laboratory of Animal Food Function, Graduate School of Agricultural Science, Tohoku University, Sendai 980-8572, Japan; yoshiya.imamura.p8@dc.tohoku.ac.jp (Y.I.); fu.namai.a3@tohoku.ac.jp (F.N.); keita.nishiyama.a6@tohoku.ac.jp (K.N.); 3 Livestock Immunology Unit, International Education and Research Centre for Food and Agricultural Immunology (CFAI), Graduate School of Agricultural Science, Tohoku University, Sendai 980-8572, Japan; 4 Department of Food, Agriculture and Environment, Miyagi University, Sendai 980-8572, Japan; suda@myu.ac.jp; 5 Gabrichevsky Research Institute for Epidemiology and Microbiology, 125212 Moscow, Russia

**Keywords:** *Corynebacterium pseudodiphtheriticum*, respiratory pathogens, microbe–microbe interaction

## Abstract

In previous studies, it was demonstrated that *Corynebacterium pseudodiphtheriticum* 090104, isolated from the human nasopharynx, modulates respiratory immunity, improving protection against infections. Here, the antagonistic effect of the 090104 strain on respiratory pathogens, including *Streptococcus pneumoniae*, *Staphylococcus aureus*, *Klebsiella pneumoniae*, *Pseudomonas aeruginosa*, and *Acinetobacter baumannii*, was explored. In a series of in vitro studies, the capacity of *C. pseudodiphtheriticum* 090104, its bacterium-like particles, and its culture supernatants to coaggregate, inhibit the growth, and change the virulent phenotype of pathogenic bacteria was evaluated. The results showed that the 090104 strain was able to exert a bacteriostatic effect on *K. pneumoniae* and *S. pneumoniae* growth. In addition, *C. pseudodiphtheriticum* 090104 coaggregated, inhibited biofilm formation, and induced phenotypic changes in all the respiratory pathogens evaluated. In conclusion, this work demonstrated that, in addition to its beneficial effects exerted by host–microbe interactions, *C. pseudodiphtheriticum* 090104 can enhance protection against respiratory pathogens through its microbe–microbe interactions. The mechanisms involved in such interactions should be evaluated in future research.

## 1. Introduction

Various studies demonstrated the ability of probiotic microorganisms to enhance resistance to intestinal infections such as those induced by *Lacticaseibacillus rhamnosus* GG against rotavirus [1,2,3]. Furthermore, diverse mechanisms through which probiotics can exert antagonistic effects on intestinal pathogens have been reported. Among them, competition for host tissue binding sites; competition for nutrients; production of metabolites affecting the phenotype of pathogens by inhibiting, for example, their growth or biofilm formation; and the modulation of intestinal immune responses have been described [1,2,3]. Through these microbe–microbe and microbe–host interactions, probiotics can help to reduce the severity of intestinal infections [4,5,6,7,8].

Microbe–microbe interactions can also affect the outcome of infections in the respiratory tract, such as the inhibition exerted by *Dolosigranulum pigrum* against the nasopharyngeal colonization of pathogenic *Staphylococcus aureus* and *Streptococcus pneumoniae* [9]. There are reports describing microbial interactions between strains of the genus *Corynebacterium* and pathogenic bacteria, which alter the virulence and/or viability of the latter. In this regard, it was demonstrated that strains of the species *Corynebacterium amycolatum* can coaggregate with pathogenic strains of *Klebsiella pneumoniae*, *Pseudomonas aeruginosa*, and *S. aureus* [10]. It was also reported that the non-pathogenic respiratory commensal bacterium *Corynebacterium accolens* can inhibit the adhesion and invasion of *S. aureus* to human nasal epithelial cells [11], while strains of the species *Corynebacterium bovis* [12], *Corynebacterium striatum* [13,14], and *Corynebacterium pseudodiphtheriticum* [15] can inhibit the growth of *S. aureus* in vitro. Corynebacteria can also exert inhibitory effects on other respiratory pathogens, as demonstrated by studies in which *S. pneumoniae* was inhibited by strains of *C. accolens* and *C. amycolatum* [16], and *A. baumannii* was inhibited by strains of *Corynebacterium aurimucosum*, *Corynebacterium aquatimens*, and *Corynebacterium mucifaciens* [17]. Interestingly, it was determined that corynebacteria can modulate the phenotype of respiratory pathogens. It was shown that *Corynebacterium xerosis* prevents biofilm formation on abiotic surfaces of *S. aureus*, *Streptococcus mutans*, *Escherichia coli*, and *P. aeruginosa* [18], while *C. striatum* reduces the hemolytic activity of *S. aureus* [13].

In previous studies, we demonstrated that the respiratory commensal bacterium *C. pseudodiphtheriticum* 090104, originally isolated from the human nasopharynx, is capable of modulating innate and adaptive immune responses in the respiratory tract of mice when nasally administered [19,20,21]. Through its capacity to modulate the function of dendritic cells, alveolar macrophages, and T cells in the respiratory tract, the 090104 strain can enhance the resistance to *S. pneumoniae* and RSV infections [19,20]. Although the microbe–host interaction has been extensively evaluated in relation to the beneficial effects of *C. pseudodiphtheriticum* 090104, its microbe–microbe interactions with relevant respiratory pathogens was not evaluated before. Then, the aim of this study was to characterize the capacity of the 090104 strain to exert antagonistic effects on respiratory pathogens of clinical importance.

## 2. Materials and Methods

### 2.1. Microorganisms

The respiratory commensal bacterium *C. pseudodiphtheriticum* 090104 (Cp090104) and its bacterium-like particles (BLPs, designated here as PCp090104) were prepared as described in previous works [19,20,21]. Six respiratory pathogens were also employed: *K. pneumoniae* LABACER27 and LABACER01 [22], *P. aeruginosa*, *S. aureus*, *A. baumannii*, and *S. pneumoniae* 19F [this work], which were isolated from different human clinical samples and preserved at the Bacteriology Laboratory of the Institute of Microbiology “Dr. Luis C. Verna” of the Faculty of Biochemistry, Chemistry, and Pharmacy—UNT (Tucumán) or at the Culture Collection of the ANLIS-Malbran Institute (Buenos Aires) (Table 1).

All bacteria were preserved and stored in 20% (*v*/*v*) glycerol at −20 °C [22]. For activation, they were cultured on 1.5% (*w*/*v*) BHI agar plates (Britania) and incubated at 37 °C under aerobic conditions for 24 h. Selected colonies were inoculated into BHI broth and cultivated at 37 °C without agitation for 18 h or until reaching a DO_600 nm_ = 0.8 and diluted according to the experiment to be performed.

In some experiments, the cell-free supernatant of *C. pseudodiphtheriticum* 090104 was used, for which the protocol of Dey et al. [23], with some modifications, was employed. Briefly, the 090104 strain was inoculated into 5 mL of BHI broth and cultivated at 37 °C for 12 h, without agitation. The culture was centrifuged for 8 min at 8000 rpm (Sorvall ST16R-Thermo Fisher, Waltham, MA, USA). The supernatant was collected with a sterile syringe and needle and filtered using a sterile polyethersulfone membrane filter with a pore size of 0.22 μm and a diameter of 25 mm (Sigma-Aldrich, St. Louis, MO, USA). The cell-free supernatant was stored at −20 °C until use.

### 2.2. Autoaggregation Test

The ability of bacteria to autoaggregate is considered essential for their adhesion to mucosal surfaces [24,25,26], making this characteristic desirable for strains of probiotic microorganisms [27]. The autoaggregation capacity of the bacteria was measured using the protocol of [28], but with slight modifications. The bacterial cultures incubated for 12 h were centrifuged at 8000 rpm (Sorvall ST16R-Thermo Fisher) for 10 min at room temperature, washed twice with PBS, and then resuspended in 3 mL of the same buffer adjusting the DO_600 nm_ to 0.3. Bacterial suspensions were incubated at room temperature and examined at different time intervals (0, 2, 4, 6, 8, 10, and 12 h). The percentage of autoaggregation (A%) was calculated as follows: A% = (A_0_ − A_t_)/A_0_ × 100, where A_0_ represents the absorbance (A_600 nm_) at time 0, and A_t_ represents the absorbance (A_600 nm_) at different time intervals.

### 2.3. Coaggregation Test

Coaggregation refers to the microbiological process by which different bacterial species join together to form aggregates. This bacteria–bacteria interaction has been described as one of the mechanisms through which probiotic microorganisms can reduce the colonization and persistence of pathogens on mucosal surfaces [25]. The coaggregation of the microorganisms was evaluated using the widely described methodology [28,29]. Bacterial suspensions were prepared in sterile PBS, as described in the autoaggregation assay, and then each of the respiratory pathogens was brought into contact with Cp090104 or PCp090104. For this, equal volumes of the respiratory commensal bacterial suspensions or their BLPs and the pathogenic bacteria (1.5 mL) were mixed in sterile tubes and incubated at room temperature, without agitation. The absorbances (A_600 nm_) of the mixtures were determined at different intervals: 0, 2, 4, 6, 8, and 10 h. Pure suspensions of Cp090104, PCp090104, and the respiratory pathogens were also used, and their absorbance was measured at the same times. Coaggregation was calculated as follows: [(A_pat_ + A_cp_)/2 − (A_mix_)]/(A_pat_ + A_cp_)/2 × 100, where A_pat_ and A_cp_ represent the A_600 nm_ of the bacterial suspensions of the pathogen and Cp090104 (or PCp090104), while A_mix_ represents the absorbance of the co-culture.

### 2.4. Inhibitory Activity Assay

To determine the ability of Cp090104 and its cell-free supernatant to inhibit the growth of respiratory pathogens, the method of Denkova et al. [30] was employed. First, 10 mL of 1.5% (*w*/*v*) BHI agar medium was mixed with a 1% (*v*/*v*) inoculum of Cp090104 (DO_600 nm_ = 0.8, equivalent to 10^8^ CFU/mL) or the cell-free supernatant, transferred to Petri dishes, and incubated for 24 h at 37 °C to allow the development of a Cp090104 lawn. In the case of the cell-free supernatant, it was also incubated for 24 h at 37 °C to discard Cp090104 growth. Then, 10 µL of each different dilution (1:1, 1:2, 1:4, and 1:8) of the pathogen suspension (DO_600 nm_ = 0.8) was inoculated onto the Cp090104 lawn, allowed to dry, and incubated at 37 °C for 24 h. Inhibitory activity was calculated considering the volume of the inoculated sample, the highest dilution at which pathogen growth was inhibited, and the growth curve of each pathogen. Inhibition was confirmed by taking a sample with a sterile swab from the area inoculated with the pathogen after exposure to Cp090401. This sample was plated on BHI agar and incubated for 48 h at 37 °C.

The same protocol was used to evaluate the inhibitory capacity of each respiratory pathogen on the growth of Cp090104. BHI agar medium was inoculated with an aliquot of the bacterial suspension of each pathogen (OD_600 nm_ = 0.8) to create the lawn and incubated for 24 h at 37 °C. Then, different dilutions (1:1, 1:2, 1:4, and 1:8) of Cp090104 (OD_600 nm_ = 0.8) were inoculated into the medium, allowed to dry, and incubated at 37 °C for 24 h.

### 2.5. Biofilm Formation Assay

Biofilm formation is a fundamental survival strategy for bacteria, allowing them to adhere to surfaces and enclose themselves in a self-produced matrix of extracellular polymeric substances [31]. Biofilms can promote colonization and resistance to antimicrobials by pathogens [32,33]. The capacity for biofilm formation of Cp090104 and the respiratory pathogens was evaluated using the methodology described in the literature [34]. Briefly, from each bacterial suspension cultured for 18 h, an inoculum (5% *v*/*v*) was incubated with 200 µL of fresh sterile BHI broth in a 96-well polystyrene plate for 24 and 48 h. This procedure was performed in triplicate. Wells containing only BHI broth were considered negative controls to exclude any effect of nonspecific binding to the staining solution. To quantify biofilm formation, after incubation, the wells were washed with PBS, and the adherent bacteria were treated for 30 min with 200 μL of 0.1% (*w*/*v*) crystal violet in an isopropanol–methanol–PBS solution (1:1:18, *v*/*v*/*v*). Excess dye was washed off with 200 µL of sterile distilled water per well. Subsequently, the dye bound to the adhered cells was extracted with 200 µL of 30% (*v*/*v*) glacial acetic acid. Then, 135 µL from each well was transferred, and the OD_570 nm_ was measured using an ELISA reader (Tecan, Männedorf, Switzerland). To classify biofilm-forming bacteria, a cutoff OD (ODc) was determined following the criteria according to Stepanović et al. [34]. ODc equals three standard deviations above the mean OD of the negative control. The OD_570 nm_ value of strains above the cutoff line was considered positive for biofilm formation. This methodology also allows the classification of bacteria under study into four groups: non-adherent (OD ≤ ODc), weakly adherent (ODc < OD ≤ 2 ODc), moderately adherent (2 ODc < OD ≤ 4 ODc), and strongly adherent (4 ODc < OD).

### 2.6. Inhibition of Biofilm Formation Assay

The ability of the cell-free supernatant of Cp090104 to inhibit the biofilm formation of respiratory pathogens was determined using the method of [35], but with modifications. Bacterial suspensions cultured for 18 h were used to obtain cell-free supernatant. Then, 10 μL of Cp090104 supernatant was added to 10 μL of the pathogen culture and 180 μL of sterile BHI broth and incubated for 24 and 48 h. This procedure was performed in triplicate. Control groups included wells containing 10 μL of respiratory pathogen suspension plus 190 μL of BHI broth, and wells with 200 μL of cell-free supernatant and 200 μL of sterile BHI broth. After incubation, the wells were treated as described above, with crystal violet. To determine the effect on biofilm formation, the formula of Khusro et al. [36] was applied: 1 − (OD_A_/OD_B_) × 100, where OD_A_ is the absorbance of the well containing the mixture of Cp090104 supernatant and the respiratory pathogen suspension, and OD_B_ is the absorbance of the well containing each respiratory pathogen.

### 2.7. Inhibition of Hemolytic Activity Assay

There is evidence that pathogenic bacteria, in the presence of other commensal or pathogenic microorganisms, can change their phenotype, which impacts their virulence [13,31,32]. To study the hemolytic activity of the microorganisms, BHI–blood agar was used [30,37], which was prepared with sterile 1.5% (*w*/*v*) BHI agar and 5% (*v*/*v*) defibrinated sheep blood. Aliquots of 10 µL of bacterial suspensions were inoculated onto the BHI–blood agar and incubated at 37 °C for 24 h, and hemolytic activity was observed and classified as α, β, or γ hemolysis. To evaluate the ability of Cp090104 to modify or inhibit the hemolytic activity of respiratory pathogens, a lawn of the respiratory commensal bacterium was prepared by mixing 10 mL of BHI–blood agar with a 1% (*v*/*v*) inoculum of Cp090104 and incubated for 24 h at 37 °C. BHI–blood agar plates with the addition of Cp090104 cell-free supernatant were also prepared and immediately used without incubation. Four 10 µL aliquots of each pathogen suspension (OD_600 nm_ = 0.8, ratio 1:1) were placed on the media containing Cp090104 or its supernatant and incubated at 37 °C for 24 h. Hemolytic activity was classified as α, β, or γ hemolysis and compared with cultures of the pathogens grown in the absence of Cp090104 or its supernatant.

### 2.8. Morphology of Respiratory Pathogen Colonies

To evaluate the effect of Cp090104 on the morphology of respiratory pathogen colonies, the method of Hossain et al. [24], but with some modifications, was used. Briefly, plates of sterile 0.3% (*w*/*v*) soft BHI agar were prepared with the addition of a 1% (*v*/*v*) inoculum of Cp090104 or its cell-free supernatant. Plates containing only sterile soft BHI agar were used as controls. Then, 5 µL aliquots of pathogen suspensions (OD_600 nm_ = 0.8) were placed in the plates, and the plates were incubated at 37 °C for 120 h. Colonies were visually analyzed, and diameters (cm) were measured. Photographic records of the colonies developed under different experimental conditions were also taken.

### 2.9. Statistical Analysis

For quantitative tests, units of measurement were defined and expressed as mean ± standard deviation (SD). Experiments were performed in duplicate or triplicate. ANOVA test was used to assess differences and significance of the obtained data.

## 3. Results

### 3.1. Study of the Autoaggregation and Coaggregation Capacity of C. pseudodiphtheriticum 090104

**Figure 1:**
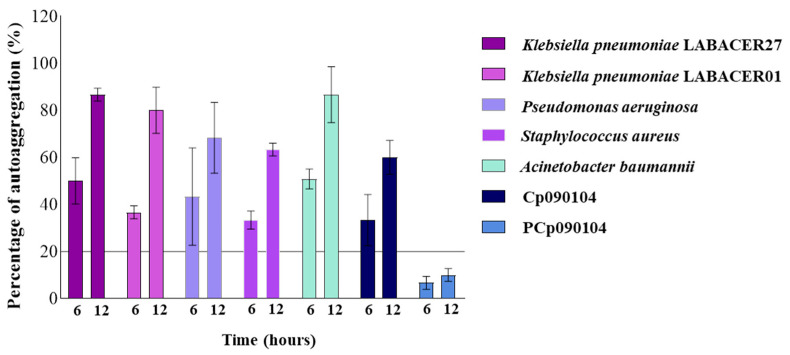
Autoaggregation assay. The autoaggregation was evaluated in distinct respiratory pathogens and *C. pseudodiphtheriticum* 090104 (Cp090104) and its BLPs (PCp090104). The cutoff value for bacterial aggregation is indicated by a solid line. Each bar corresponds to a time point of the assay, depicting the percentage of autoaggregation at 6 and 12 h. All tests were performed in triplicate and in three independent experiments (*n* = 9). Means with standard deviations are presented for each measurement.

The autoaggregation capacity of *C*. *pseudodiphtheriticum* 090104 and its BLPs was evaluated and compared with different respiratory pathogens (Figure 1). It was observed that Cp090104 showed an autoaggregation percentage of approximately 30% at 6 h, reaching 60% after 12 h of incubation. As expected, PCp090104 showed significantly lower aggregation percentages compared to those found in Cp090104, reaching only 10% at 12 h (Figure 1). The respiratory pathogens evaluated all achieved autoaggregation percentages higher than those found for Cp090104, especially *K. pneumoniae* LABACER01, LABACER27, and *A. baumannii*, which surpassed 80% after 12 h of incubation (Figure 1). Considering the criterion of [25], which considers strains strongly autoaggregative if they reach or exceed 80%, and non-autoaggregative if they present values equal to or less than 10%, Cp090104 can be considered a bacterium with weak autoaggregative ability.

**Figure 2:**
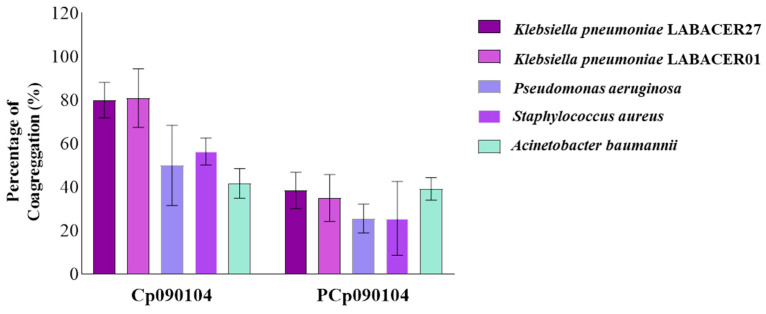
Coaggregation assay. The coaggregation assay was performed with *C. pseudodiphtheriticum* 090104 (Cp090104) and its BLPs (PCp090104) and the distinct respiratory pathogens. Bars for each respiratory pathogen represent the sampling time at 10 h (endpoint). All tests were performed in triplicate and in three independent experiments (*n* = 9). Means with standard deviations are presented for each measurement.

The ability of Cp090104 and PCp090104 to coaggregate with the different respiratory pathogens was also studied (Figure 2).

After 10 h of incubation of Cp090104 with the pathogenic strains, a notable capacity to coaggregate with *K. pneumoniae* LABACER01 and LABACER27 was observed, reaching coaggregation percentages of approximately 80% in both cases. Conversely, the lowest coaggregation percentage was detected for *A. baumannii*, with only 41% (Figure 2). When evaluating the coaggregation ability of PCp090104 with respiratory pathogens, percentages were approximately half the value of those found with Cp090104, except for *A. baumannii*, which showed similar values to those found with the live respiratory commensal bacterium (Figure 2).

### 3.2. Evaluation of C. pseudodiphtheriticum 090104 Capacity to Inhibit the Growth of Pathogens

**Figure 3:**
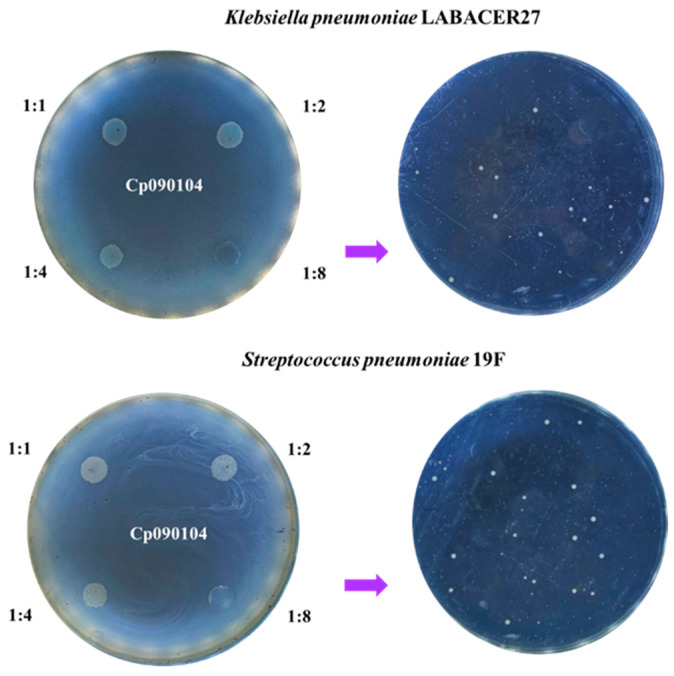
Inhibitory activity assay. Inhibition with spot inoculation of two respiratory pathogens on *C. pseudodiphtheriticum* 090104 (Cp090104) lawn. Dilutions made for both pathogens are indicated. Photos on the right show the passage of residual inoculum after contact with Cp090104. All assays were performed in duplicates.

Next, the ability of *C. pseudodiphtheriticum* 090104 to inhibit the growth of respiratory pathogens was evaluated. In this assay, the interaction between pathogenic bacteria and Cp090104 was evaluated using agar medium containing the respiratory commensal bacterium, to which the pathogens were added (Figure 3). In this method, a concentration of Cp090104 equal to or greater than (1:1, 1:2, 1:4, and 1:8) that of the microorganisms to be tested is used, simulating a previous colonization of the respiratory commensal bacterium at the contact site with the pathogens. An inhibitory effect by Cp090104 on *K. pneumoniae* LABACER27 and *S. pneumoniae* 19F was observed when they were inoculated at a 1:8 dilution (Figure 3).

This inhibition was partial, as the development of *K. pneumoniae* LABACER27 and *S. pneumoniae* 19F was observed after swabbing the surface of the 1:8 dilution following contact with Cp090104 (Figure 3), indicating a bacteriostatic rather than bactericidal effect. The inhibition study using growth curves of the pathogens allowed us to determine that *K. pneumoniae* LABACER27 is inhibited by 10^8^ cells of Cp090104 at doses less than or equal to 10^3^ CFU/mL, while *S. pneumoniae* 19F is inhibited by doses less than or equal to 10^4^ CFU/mL. On the other hand, *C. pseudodiphtheriticum* 090104 was not able to inhibit the growth *of K. pneumoniae* LABACER01, *P. aeruginosa*, *S. aureus*, or *A. baumannii*. Additionally, the potential inhibitory effect of the cell-free supernatant of Cp090104 was studied, and no bactericidal or bacteriostatic effects were observed on any of the respiratory pathogens evaluated.

**Figure 4:**
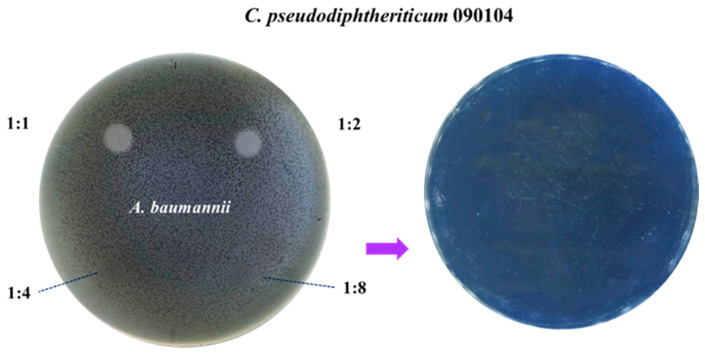
Inhibitory activity assay. Inhibition with spot inoculation of *C. pseudodiphtheriticum* 090104 (Cp090104) on *A. baumannii* lawn. Dilutions made for the respiratory commensal bacterium are indicated. Photos on the right show the passage of residual inoculum after contact with *A. baumannii*. All assays were performed in duplicates.

The potential inhibitory effect of the respiratory pathogens on *C. pseudodiphtheriticum* 090104 was also evaluated (Figure 4). For this study, the respiratory commensal bacterium was cultured on agar plates previously inoculated with the different pathogens. No inhibitory effects on Cp090104 were observed when *K. pneumoniae* LABACER01 and LABACER27, *P. aeruginosa*, *S. aureus*, or *S. pneumoniae* 19F were studied. Conversely, *A. baumannii* was able to inhibit the growth of Cp090104 from a 1:4 dilution (Figure 4). This inhibition was complete, as no development of Cp090104 was observed after swabbing the surface of the 1:4 dilution following contact with *A. baumannii* (Figure 4), indicating a bactericidal effect. The inhibition study using growth curves of the respiratory commensal bacterium allowed us to determine that it is inhibited by *A. baumannii* at doses less than or equal to 10^4^ CFU/mL.

### 3.3. Study of C. pseudodiphtheriticum 090104 Capacity to Form and Inhibit Biofilms

**Figure 5:**
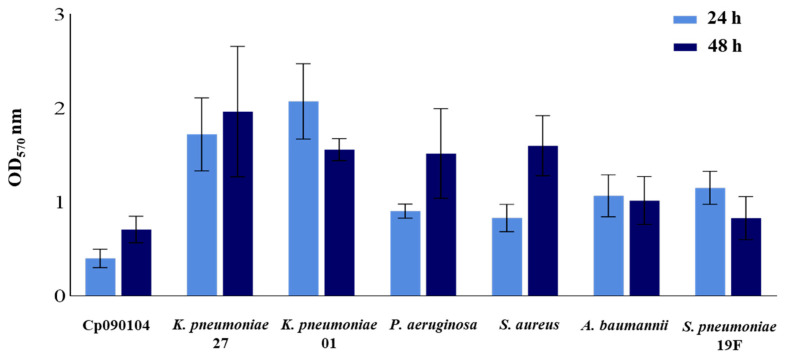
Biofilm formation assay. Biofilm formation assay of distinct respiratory pathogens and *C. pseudodiphtheriticum* 090104 (Cp090104). Two sampling times are presented, 24 and 48 h. All tests were performed in triplicate and in three independent experiments (*n* = 9). Means with standard deviations are presented for each measurement.

The capacity of Cp090104 to form biofilms on an abiotic surface was studied and compared with respiratory pathogens (Figure 5). Under the experimental conditions of this work, all the pathogenic microorganisms were able to form biofilms after 48 h of incubation, with *K. pneumoniae* LABACER01 and LABACER27 being the most efficient in achieving this effect. Taking into account Stepanović et al.’s classification [34], which considers the variation in biofilm formation between 24 and 48 h, respiratory pathogens were classified as weakly adherent (*A. baumannii*, *P. aeruginosa*, and *S. pneumoniae* 19F) or moderately adherent (*K. pneumoniae* LABACER27 and LABACER01, and *S. aureus*). On the other hand, the results showed that Cp090104 was not able to form a biofilm on the abiotic surface and exhibited weak adherence (Figure 5).

**Figure 6:**
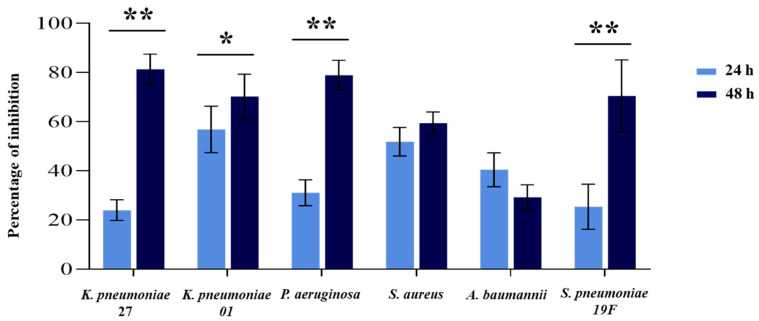
Inhibition of biofilm formation assay. Study of the effect of the cell-free supernatant of *C. pseudodiphtheriticum* 090104 (Cp090104) on biofilm formation by the respiratory pathogens. All tests were performed in triplicate and in three independent experiments (*n* = 9). Means with standard deviations are presented for each measurement. Significant differences between the indicated groups: (*) *p* < 0.05; (**) *p* < 0.01.

Additionally, the ability of the cell-free supernatant of Cp090104 to inhibit biofilm formation by different respiratory pathogens was evaluated (Figure 6). After 24 h of incubation, biofilm formation had at least a 20% of inhibition for all microorganisms, with *K. pneumoniae* LABACER27 and LABACER01 being the most and least inhibited biofilm-forming pathogens, respectively (Figure 6). After 48 h of incubation, the supernatant of Cp090104 exerted a greater inhibitory effect on biofilm formation in all bacteria except *A. baumannii* and *S. aureus*, which remained at similar percentages to those found at 24 h (Figure 6).

### 3.4. Effect of C. pseudodiphtheriticum 090104 on the Phenotype of Respiratory Pathogens

Finally, whether Cp090104 can induce changes in the phenotype of respiratory pathogens was evaluated, and hemolytic activity was selected for this purpose. Firstly, the hemolytic activities of the six pathogens used in this work were studied (Table 2). Assays conducted on BHI agar–blood allowed for the detection of different types of hemolytic activity in respiratory pathogens: γ hemolysis for *K. pneumoniae* LABACER27 and LABACER01, and *A. baumannii*; β hemolysis for *S. aureus* and *P. aeruginosa*; and α hemolysis for *S. pneumoniae.*

**Figure 7:**
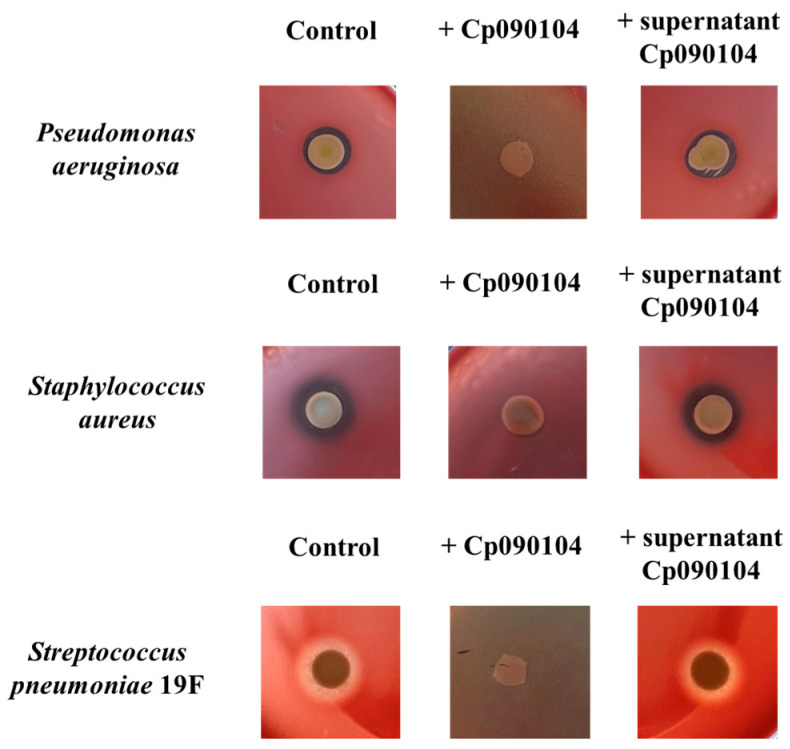
Hemolytic activity assay. Characterization of the effect of *C. pseudodiphtheriticum* 090104 (Cp090104) and its cell-free supernatant on the hemolytic activity of respiratory pathogens when cultured on BHI agar–blood medium. Tests were performed in triplicate and in three independent experiments.

When pathogens were cultured on blood agar in the presence of Cp090104, a loss of hemolytic activity was detected for *P. aeruginosa*, *S. aureus*, and *S. pneumoniae* 19F (Table 2 and Figure 7). No changes in hemolytic activity were observed for *K. pneumoniae* LABACER27 and LABACER01, and *A. baumannii* in the presence of Cp090104. Additionally, it was observed that the cell-free supernatant of Cp090104 did not induce changes in the hemolytic activity of any of the evaluated pathogenic microorganisms (Table 2 and Figure 7).

**Figure 8:**
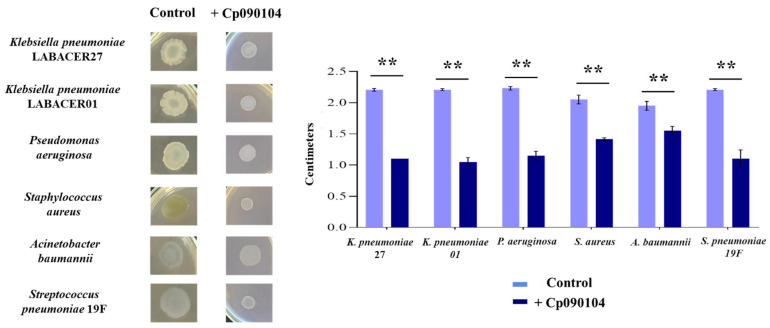
Colony morphology assay. Characterization of the effect of *C. pseudodiphtheriticum* 090104 (Cp090104) on the colony morphology of respiratory pathogens when cultured on BHI agar medium. The diameter of colony was measured in the presence and absence of Cp090104. All tests were performed in triplicate; means with standard deviations are presented for each measurement. Significant differences between the indicated groups: (**) *p* < 0.01.

To corroborate that *C. pseudodiphtheriticum* 090104 can induce changes in the phenotype of respiratory pathogens, the morphology of their colonies was compared in the presence and absence of the respiratory commensal bacterium after 120 h of incubation. As shown in Figure 8, the mucoid morphologies of the colonies of *P. aeruginosa*, *K. pneumoniae* LABACER27 and LABACER01, and *S. pneumoniae* 19F were not observed in the presence of Cp090104. In all cases, the colonies had more defined edges and were smaller in size. A similar but less pronounced effect was detected when comparing the colonies of *A. baumannii* cultured in the absence and presence of Cp090104 (Figure 8). On the other hand, the yellowish colony of *S. aureus* decreased in size and turned white when the pathogen was cultured in the presence of Cp090104 (Figure 8). When the cell-free supernatant of Cp090104 was used, no phenotypic changes were observed in the colonies of any of the respiratory pathogens studied.

## 4. Discussion

In previous works, we demonstrated the ability of the commensal bacterium *C. pseudodiphtheriticum* 090104 to modulate respiratory immunity, resulting in decreased susceptibility to infections [19,20,21]. In this work, we further characterized the probiotic effects of the strain 090104 by showing that this bacterium could exert antagonistic effects on various respiratory pathogens.

The autoaggregation of bacteria is considered important for their attachment to epithelial surfaces, as they provide an initial anchorage that allows them to colonize host mucosa [26,38]. Autoaggregation is advantageous for bacteria, as it contributes to their resistance to external pressures. For example, autoaggregation was reported to help bacteria to protect themselves when facing nutrient scarcity or oxidative stress. It can also contribute to protecting them from the immune system [26]. Therefore, this property was described in numerous pathogens, including those infecting the respiratory tract. In this regard, the capacity for autoaggregation has been reported for *P. aeruginosa*, *S. aureus*, and *A. baumannii* strains [26], as well as for carbapenemase-producing *K. pneumoniae* strains [39], which use it to initiate their infection cycle. Consistent with these previous reports, it was observed that all the respiratory pathogens studied here exhibited autoaggregating capacities, which were particularly notable for *K. pneumoniae* LABACER27, LABACER01, and *A. baumannii*. Studies have also described autoaggregative capabilities in pathogenic species of *Corynebacterium* [40,41]; however, this behavior has not been thoroughly studied for *C. pseudodiphtheriticum* strains.

A study conducted with *C. pseudodiphtheriticum* ATCC10700, isolated from exudative pharyngitis, described an adherence pattern called aggregative adherence, characterized by groups of bacteria with a “stacked brick” appearance upon contact with human HEp-2 epithelial cells [42]. The work also observed that HEp-2 cells were infected by the ATCC10700 strain, and then the autoaggregation of *C. pseudodiphtheriticum* was associated with its virulence resembling other respiratory pathogens. In contrast, when *C. pseudodiphtheriticum* 090104 was studied here, a weak/moderate autoaggregating ability was found according to the criteria of Balakrishna et al. [25]. The low/moderate autoaggregating capacity of the 090104 strain could be associated with its lack of virulence and might not be associated with its ability to colonize respiratory mucosa, as described for other commensal bacteria. It was reported that *C. accolens* (strains C779, C781, and C787), another species of non-pathogenic respiratory commensal bacteria, has a high capacity to adhere to human nasal epithelial cells and prevent invasion by *S. aureus* [11]. It was also described that such adherence to host cells was not related to the autoaggregating capacity of the strains. Another example is the study describing that *C. amycolatum* (strains ICIS 9 and ICIS 53), isolated from the vaginal mucosa of healthy women, exhibits a moderately autoaggregative phenotype despite its high capacity to adhere to epithelial cells [43]. It is necessary to consider that the autoaggregative capacity of bacteria can be altered in response to modifications in their environment, such as changes in oxygen levels or temperature variations [26,38], scenarios that can occur along the host’s respiratory tract. Therefore, further studies of the 090104 strain are needed to evaluate whether its autoaggregating capacity varies upon contact with host cells and under environmental conditions that simulate those of the respiratory tract.

The ability of bacteria to aggregate is closely related to their capacity to form biofilms, as the former is an initial step in the ecological adaptation that culminates in the establishment of a biofilm and subsequent colonization of the microbial community on a biotic or abiotic surface [44,45]. Thus, pathogenic bacteria, including respiratory ones [46,47,48,49], can form biofilms on a wide variety of surfaces, such as living tissues and medical devices [31,50,51]. The capacity for biofilm formation has been documented in *Corynebacterium* species [52,53], a property that is pronounced in pathogenic species such as *C. jeikeium*, *C. macginleyi*, and *C. diphtheriae* [54,55,56]. This property was also described for *C. pseudodiphtheriticum* strains associated with infections. *C. pseudodiphtheriticum* HHC1507, isolated from a patient’s blood with systemic infection, and *C. pseudodiphtheriticum* ATCC10700, isolated from the respiratory tract of a patient with pharyngitis, showed high and moderate abilities to form biofilms on plastic surfaces, respectively [57]. In line with their autoaggregating capacities, the respiratory pathogens studied here exhibited a marked ability to form biofilms that was greater than that observed in *C. pseudodiphtheriticum* 090104. The low autoaggregating and biofilm-forming capacities of the strain 090104 would not affect its adhesion to the respiratory epithelium or its ability to modulate the immune system in vivo, as its BLPs, which preserve the immunomodulatory properties of live bacteria, did not exhibit autoaggregating abilities and, of course, were unable to form a biofilm.

Although *C. pseudodiphtheriticum* 090104 exhibited low/moderate autoaggregation, it was observed that this respiratory commensal bacterium could coaggregate with all the respiratory pathogens studied. These results are in line with reports describing the ability of *C. amycolatum* ICIS 9 and *C. amycolatum* ICIS 53 to coaggregate with pathogenic strains of *K. pneumoniae*, *P. auruginosa*, and *S. aureus* [10]. It was reported that the coaggregation property of probiotics can play an important role in limiting the virulence of pathogenic bacteria by inhibiting their adherence and colonization in the host mucosa [58,59]. Additionally, coaggregation enhances other probiotic properties, such as competition for nutrients, inhibition by antimicrobial substances, and the modulation of virulence factors’ expression by favoring closer contacts between microorganisms [60].

Studies have evaluated the ability of probiotic microorganisms to coaggregate with respiratory pathogens, and it has been reported that this property depends on the strain analyzed, the bacterial pathogen, and the incubation conditions [28]. *L. plantarum* MBS17 [61] and *L. fermentum* LP10 [62] have been described to coaggregate with pathogenic strains of *K. pneumoniae*, while *L. brevis* gp104 can coaggregate with *S. aureus* [63]. This phenomenon has also been reported with pathogenic strains of *P. aeruginosa* isolated from sputum samples of cystic fibrosis patients, which can coaggregate with lactobacilli and bifidobacteria strains [64,65]. On the other hand, it was documented that *B. subtilis* KATMIRA1933 and *B. amyloliquefaciens* B-1895 are capable of coaggregating with various *Acinetobacter* spp. isolates [66]. There is no published precedent describing the ability of a strain of the species *C. pseudodiphtheriticum* to coaggregate with respiratory pathogens, making this work the first report in that regard.

One of the most commonly used tests in selecting probiotic microorganisms for prevention or treatment of infections is to determine their ability to inhibit the growth of pathogens in vitro [1,2,3]. Therefore, the potential inhibitory effect of *C. pseudodiphtheriticum* 090104 against all the respiratory pathogens was also evaluated. The experiments showed that the respiratory commensal bacterium managed only to inhibit the growth of *K. pneumoniae* LABACER27 and *S. pneumoniae* 19F, and that this effect was bacteriostatic rather than bactericidal. These results could explain data obtained in vivo, where *C. pseudodiphtheriticum* 090104 was used to enhance the resistance to respiratory infections produced by nasal challenges with *K. pneumoniae* LABACER27 and LABACER01 [67]. The treatment of mice with the respiratory commensal bacterium before the challenge with LABACER27 and LABACER01 strains increased the resistance to infections, evidenced by lower bacterial loads in lungs and blood, decreased lung damage, and improved survival rates compared to control mice. This effect was associated with the ability of the strain 090104 to modulate respiratory and systemic innate immunity. It is worth noting that the protective effect of *C. pseudodiphtheriticum* 090104 was greater in the infection by *K. pneumoniae* LABACER27 compared to that of the LABACER01 strain [67]. It is possible to speculate that this difference is due to the bacteriostatic effect exerted by the 090104 strain on *K. pneumoniae* LABACER27, allowing the immune system to act more efficiently. We hypothesized that genomic differences between LABACER27 and LABACER01 strains [22,68] could explain the higher virulence of the latter in the in vivo model, as well as the lower efficacy in the protection induced by *C. pseudodiphtheriticum* 090104. The results obtained here reinforce this hypothesis since genomic differences between the strains could also explain the lack of inhibitory effect of the respiratory commensal bacterium on *K. pneumoniae* LABACER01.

On the other hand, the bacteriostatic effect of *C. pseudodiphtheriticum* 090104 on *S. pneumoniae* would also contribute to protection against pneumococcal infection in vivo in addition to its immunomodulatory effect. Consistent with our results, it was reported that bacterial lipases produced by strains of *C. accolens* and *C. amycolatum* are essential for inhibiting the growth of *S. pneumoniae* in vitro [16]. Furthermore, using a murine model, it was described that the colonization of the nasopharynx by *C. accolens* or *C. amycolatum* significantly decreased the colonization of *S. pneumoniae* in the upper respiratory tract and reduced lung infection. Additionally, mice treated with these *Corynebacterium* species exhibited a respiratory immune response regulated differentially and characterized by reduced inflammatory damage [16]. The results of previous studies [19,20,21] and those presented here suggest that the protective effects exerted by commensal Corynebacteria are mediated by mechanisms dependent and independent of the modulation of the immune system, which could act together to improve resistance to infections.

Various studies have described the antagonistic effect of *Corynebacterium* spp. on the pathogen *S. aureus*. Studies conducted in the 1980s already described the ability of *C. bovis* to inhibit the growth of *S. aureus* in vitro [12]. It was also reported that *C. striatum* strains can exert a bactericidal effect on this pathogenic bacterium [13,14]. However, when the interaction of *C. pseudodiphtheriticum* 090104 with *S. aureus* was studied, no inhibitory effect was detected. This contrasts with previous studies in humans in which *C. pseudodiphtheriticum* 090104, administered in a saline suspension as an aerosol to subjects under conditions of hyperthermia and contaminated air, was able to eliminate or reduce the counts of *S. aureus* from the upper respiratory tract [69]. Two hypotheses could explain these differences, which are not mutually exclusive. One possibility is that the beneficial effect observed in humans is due to the modulation of respiratory immunity and not to a direct effect on the pathogen. Another possibility is that, under the experimental conditions of the in vitro study, *C. pseudodiphtheriticum* 090104 does not express the factor(s) that inhibit the growth of *S. aureus* in vivo. In support of this latter hypothesis, it was described that *C. pseudodiphtheriticum* USU1, isolated from the nasal mucosa of a healthy volunteer, has an in vitro inhibitory activity on *S. aureus* that is notably greater than that observed for *C. pseudodiphtheriticum* 10700, a laboratory-adapted strain [15]. Further studies are needed to determine the mechanism(s) by which *C. pseudodiphtheriticum* 090104 would exert its protective effect against *S. aureus* in vivo.

There are precedents for the inhibitory effect of *C. aurimucosum*, *C. aquatimens*, and *C. mucifaciens* strains on *A. baumannii* [17]. It was also reported that *A. baumannii* 17,978 inhibits the skin bacterium *C. striatum* ATCC6940 through the production of acinetobactin [70]. However, the interaction between this pathogen and *C. pseudodiphtheriticum* has not been studied. The results presented in this work show that *C. pseudodiphtheriticum* 090104 does not inhibit the growth of *A. baumannii*. On the contrary, *A. baumannii* exerts a bactericidal effect on the respiratory commensal bacterium. This raises an interesting question about whether it is possible to use *C. pseudodiphtheriticum* 090104 to increase resistance to respiratory infections produced by *A. baumannii*.

There is evidence of alterations in the genetic expression of bacteria when a microbe–microbe interaction is established, which can translate into phenotypic changes [32]. One example of these changes is the demonstrated ability of some probiotic strains to inhibit biofilm formation in pathogenic bacteria [71,72,73]. This property has also been reported in some *Corynebacterium* strains. A study demonstrated that *C. xerosis* NS5 produces a lipopeptide biosurfactant called coryxin, which prevents biofilm formation on abiotic surfaces in *S. aureus*, *S. mutans*, *E. coli*, and *P. aeruginosa* [18]. On the other hand, the cell-free supernatant of *C. amycolatum* ICIS 99 reduced biofilm formation in clinical isolates of *P. aeruginosa* and *K. pneumoniae* [43]. It has also been shown that the exposure of *S. aureus* to *C. striatum* CFCM induced a negative regulation of genes under the control of the quorum sensing detection system Agr (AgrQS), resulting in changes in the pathogenic bacterium, such as a notable reduction in its hemolytic activity [13]. Recently, it was described that *C. pseudodiphtheriticum* USU1 negatively regulates the sensor protein agrC of the Agr QS system, changing the expression of various virulence factors in *S. aureus*, which also induced changes in cell surface morphology [14,15]. Considering these precedents, the ability of *C. pseudodiphtheriticum* 090104 to induce phenotypic changes in respiratory pathogens was studied. The cell-free supernatant of *C. pseudodiphtheriticum* 090104 reduced the formation of biofilms in all respiratory pathogens studied. It was also observed that the respiratory commensal bacterium can induce changes in colony morphologies and inhibit the hemolytic activity of respiratory pathogens producing α (*S. pneumoniae* 19F) and β (*S. aureus*, *P. aeruginosa*) hemolysis. Further studies are needed to delve into the mechanisms associated with the phenotypic changes resulting from the interaction between *C. pseudodiphtheriticum* 090104 and the respiratory pathogens.

## 5. Conclusions

In conclusion, this work demonstrated that, in addition to its immunomodulatory properties, *C. pseudodiphtheriticum* 090104 has antagonistic effects on respiratory pathogens through its ability to coaggregate, inhibit their growth, interfere with biofilm formation, and alter their phenotype. The mechanisms involved in such microbe–microbe interactions should be studied in greater depth, using biochemical and molecular biology tools. For example, a detailed genetic analysis would be critical in understanding the bacteriostatic effects rather than the bactericidal effects observed in the experiments with *K. pneumoniae* and *S. pneumoniae*. In addition, the impact of these microbe–microbe interactions on the pathogens’ virulence and infectivity capacities should be evaluated in vivo.

## Figures and Tables

**Table 1:** Source of isolation and antimicrobial resistance of respiratory pathogens strains used in this work. ^1^ MLS: macrolide–lincosamide–streptogramin class.

Strain	Isolation	Resistance
*K. pneumoniae* LABACER27	Bronchial lavage (pneumonia)—Padilla Hospital [22]	β-lactams
*K. pneumoniae* LABACER01	Osteoarticular tissue (sepsis)—Padilla Hospital [22]	β-lactams, quinolones, gentamicin
*P. aeruginosa*	Sputum (pneumonia)—Padilla Hospital [this work]	Imipenem
*S. aureus*	Sputum (pharyngitis)—East Hospital [this work]	β-lactams, quinolones, aminoglycosides
*A. baumannii*	Sputum (pneumonia)—East Hospital [this work]	β-lactams, quinolones, aminoglycosides
*S. pneumoniae* 19F	Sputum (pneumonia)—Malbrán Institute [this work]	MLS ^1^, tetracycline, vancomycin

**Table 2:** Hemolytic activity assay. Types of hemolysis produced by respiratory pathogens on BHI agar–blood medium. Characterization of the effect of *C. pseudodiphtheriticum* 090104 (Cp090104) and its cell-free supernatant on the hemolytic activity of pathogens. Tests were performed in triplicate and in three independent experiments.

Pathogen	Type of Hemolysis	Hemolysis in the Presence of	
		Cp090104	Supernatant
*K. pneumoniae* LABACER27	γ	γ	γ
*K. pneumoniae* LABACER01	γ	γ	γ
*P. aeruginosa*	β	No activity	β
*S. aureus*	β	No activity	β
*A. baumannii*	γ	γ	γ
*S. pneumoniae* 19F	α	No activity	α

## Data Availability

The data presented in this study are available throughout the article.

## References

[1] Van Zyl W.F., Deane S.M., Dicks L.M. (2020). Molecular insights into probiotic mechanisms of action employed against intestinal pathogenic bacteria. Gut Microbes.

[2] Jalalifar S., Mirzaei R., Motallebirad T., Razavi S., Talebi M. (2022). The emerging role of probiotics and their derivatives against biofilm-producing MRSA: A scoping review. BioMed Res. Int..

[3] Mazziotta C., Tognon M., Martini F., Torreggiani E., Rotondo J.C. (2023). Probiotics mechanism of action on immune cells and beneficial effects on human health. Cells.

[4] Ouwehand A.C., Salminen S., Isolauri E. (2002). Probiotics: An overview of beneficial effects. Lactic Acid Bacteria: Genetics, Metabolism and Applications: Proceedings of the Seventh Symposium on Lactic Acid Bacteria: Genetics, Metabolism and Applications, 1–5 September 2002, Egmond aan Zee, The Netherlands.

[5] Collinson S., Deans A., Padua-Zamora A., Gregorio G.V., Li C., Dans L.F., Allen S.J. (2020). Probiotics for treating acute infectious diarrhoea. Cochrane Database Syst. Rev..

[6] Villena J., Li C., Vizoso-Pinto M.G., Sacur J., Ren L., Kitazawa H. (2021). *Lactiplantibacillus plantarum* as a potential adjuvant and delivery system for the development of SARS-CoV-2 oral vaccines. Microorganisms.

[7] Milner E., Stevens B., An M., Lam V., Ainsworth M., Dihle P., Stearns J., Dombrowski A., Rego D., Segars K. (2021). Utilizing probiotics for the prevention and treatment of gastrointestinal diseases. Front. Microbiol..

[8] Fiocchi A., Cabana M.D., Mennini M. (2022). Current use of probiotics and prebiotics in allergy. J. Allergy Clin. Immunol. Pract..

[9] Brugger S.D., Eslami S.M., Pettigrew M.M., Escapa I.F., Henke M.T., Kong Y., Lemon K.P. (2020). Dolosigranulum pigrum cooperation and competition in human nasal microbiota. mSphere.

[10] Gladysheva I.V., Cherkasov S.V. (2023). Antibiofilm activity of cell-free supernatants of vaginal isolates of *Corynebacterium amycolatum* against *Pseudomonas aeruginosa* and *Klebsiella pneumoniae*. Arch. Microbiol..

[11] Menberu M.A., Cooksley C., Ramezanpour M., Bouras G., Wormald P.J., Psaltis A.J., Vreugde S. (2022). In vitro and in vivo evaluation of probiotic properties of *Corynebacterium accolens* isolated from the human nasal cavity. Microbiol. Res..

[12] Hogan J.S., Pankey J.W., Duthie A.H. (1987). Growth responses of *Staphylococcus aureus* and *Streptococcus agalactiae* to *Corynebacterium bovis* metabolites. J. Dairy Sci..

[13] Ramsey M.M., Freire M.O., Gabrilska R.A., Rumbaugh K.P., Lemon K.P. (2016). *Staphylococcus aureus* shifts toward commensalism in response to *Corynebacterium* species. Front. Microbiol..

[14] Hardy B.L., Dickey S.W., Plaut R.D., Riggins D.P., Stibitz S., Otto M., Merrell D.S. (2019). *Corynebacterium pseudodiphtheriticum* exploits *Staphylococcus aureus* virulence components in a novel polymicrobial defense strategy. MBio.

[15] Hardy B.L., Bansal G., Hewlett K.H., Arora A., Schaffer S.D., Kamau E., Merrell D.S. (2020). Antimicrobial activity of clinically isolated bacterial species against *Staphylococcus aureus*. Front. Microbiol..

[16] Horn K.J., Jaberi Vivar A.C., Arenas V., Andani S., Janoff E.N., Clark S.E. (2022). *Corynebacterium* species inhibit *Streptococcus pneumoniae* colonization and infection of the mouse airway. Front. Microbiol..

[17] Shamsuzzaman M., Dahal R.H., Kim S., Kim J. (2023). Genome insight and probiotic potential of three novel species of the genus *Corynebacterium*. Front. Microbiol..

[18] Dalili D., Amini M., Faramarzi M.A., Fazeli M.R., Khoshayand M.R., Samadi N. (2015). Isolation and structural characterization of Coryxin, a novel cyclic lipopeptide from *Corynebacterium xerosis* NS5 having emulsifying and anti-biofilm activity. Colloids Surf. B Biointerfaces.

[19] Ortiz Moyano R., Raya Tonetti F., Tomokiyo M., Kanmani P., Vizoso-Pinto M.G., Kim H., Quilodrán-Vega S., Melnikov V., Alvarez S., Takahashi H. (2020). The ability of respiratory commensal bacteria to beneficially modulate the lung innate immune response is a strain dependent characteristic. Microorganisms.

[20] Kanmani P., Clua P., Vizoso-Pinto M.G., Rodriguez C., Alvarez S., Melnikov V., Takahashi H., Kitazawa H., Villena J. (2017). Respiratory commensal bacteria *Corynebacterium pseudodiphtheriticum* improves resistance of infant mice to respiratory syncytial virus and *Streptococcus pneumoniae* superinfection. Front. Microbiol..

[21] Ortiz Moyano R., Raya Tonetti F., Fukuyama K., Elean M., Tomokiyo M., Suda Y., Melnikov V., Kitazawa H., Villena J. (2023). The respiratory commensal bacterium *Corynebacterium pseudodiphtheriticum* as a mucosal adjuvant for nasal vaccines. Vaccines.

[22] Jure M.A., Albarracin L., Vargas J.M., Maidana S.D., Zamar J.C., Kitazawa H., Villena J. (2021). Draft genome sequences of two hypermucoviscous carbapenem-resistant ST25 *Klebsiella pneumoniae* strains causing respiratory and systemic infections. J. Glob. Antimicrob. Resist..

[23] Dey D.K., Khan I., Kang S.C. (2019). Anti-bacterial susceptibility profiling of *Weissella confusa* DD_A7 against the multidrug-resistant ESBL-positive *E. coli*. Microb. Pathog..

[24] Hossain M.I., Mizan M.F.R., Roy P.K., Nahar S., Toushik S.H., Ashrafudoulla M., Ha S.D. (2021). *Listeria monocytogenes* biofilm inhibition on food contact surfaces by application of postbiotics from *Lactobacillus curvatus* B. 67 and *Lactobacillus plantarum* M. 2. Food Res. Int..

[25] Balakrishna A. (2013). In vitro evaluation of adhesion and aggregation abilities of four potential probiotic strains isolated from guppy (*Poecilia reticulata*). Braz. Arch. Biol. Technol..

[26] Trunk T., Khalil H.S., Leo J.C. (2018). Bacterial autoaggregation. AIMS Microbiol..

[27] Thomas C.M., Versalovic J. (2010). Probiotics-host communication: Modulation of signaling pathways in the intestine. Gut Microbes.

[28] Collado M.C., Meriluoto J., Salminen S. (2008). Adhesion and aggregation properties of probiotic and pathogen strains. Eur. Food Res. Technol..

[29] Jena P.K., Trivedi D., Thakore K., Chaudhary H., Giri S.S., Seshadri S. (2013). Isolation and characterization of probiotic properties of lactobacilli isolated from rat fecal microbiota. Microbiol. Immunol..

[30] Denkova R., Goranov B., Teneva D., Denkova Z., Kostov G. (2017). Antimicrobial activity of probiotic microorganisms: Mechanisms of interaction and methods of examination. Antimicrob. Res. Nov. Bioknowl. Educ. Programs.

[31] Donlan R.M. (2002). Biofilms: Microbial life on surfaces. Emerg. Infect. Dis..

[32] Jałowiecki Ł., Żur J., Chojniak J., Ejhed H., Płaza G. (2018). Properties of antibiotic-resistant bacteria isolated from onsite wastewater treatment plant in relation to biofilm formation. Curr. Microbiol..

[33] Vidhya S.K. (2019). Isolation and Phenotypic Characterisation of Bacterial Isolates from Catheter Related Blood Stream Infections in Patients on Hemodialysis in a Tertiary Care Hospital. https://oa.mg/work/2997926051.

[34] Stepanovic S., Vukovic D., Dakic I., Savic B., Svabic-Vlahovic M. (2000). Modified microtiter-plate test for quantification of staphylococcal biofilm formation. J. Microbiol. Methods.

[35] Pérez Ibarreche M.P., Castellano P., Vignolo G. (2000). Evaluation of anti-Listeria meat borne Lactobacillus for biofilm formation on selected abiotic surfaces. Meat Sci..

[36] Khusro A., Aarti C., Barbabosa-Pilego A., Rojas Hernandez S. (2019). Anti-pathogenic, antibiofilm, and technological properties of fermented food associated *Staphylococcus succinus* strain AAS2. Prep. Biochem. Biotechnol..

[37] Faris Y., Atya A. (2023). Probiotic characteristics of *Enterococcus* spp. Bacteria isolated from different sources. Egypt. J. Hosp. Med..

[38] Mazlumi A., Panahi B., Hejazi M.A., Nami Y. (2022). Probiotic potential characterization and clustering using unsupervised algorithms of lactic acid bacteria from saltwater fish samples. Sci. Rep..

[39] Chiarelli A., Cabanel N., Rosinski-Chupin I., Zongo P.D., Naas T., Bonnin R.A., Glaser P. (2020). Diversity of mucoid to non-mucoid switch among carbapenemase-producing *Klebsiella pneumoniae*. BMC Microbiol..

[40] Ott L. (2018). Adhesion properties of toxigenic corynebacteria. AIMS Microbiol..

[41] Ott L., Möller J., Burkovski A. (2022). Interactions between the re-emerging pathogen *Corynebacterium diphtheriae* and host cells. Int. J. Mol. Sci..

[42] Souza M.C.D., Santos L.S.D., Gomes D.L.R., Sabbadini P.S., Santos C.S.D., Camello T.C.F., Guaraldi A.L.D.M. (2012). Aggregative adherent strains of *Corynebacterium pseudodiphtheriticum* enter and survive within HEp-2 epithelial cells. Memórias Do Inst. Oswaldo Cruz.

[43] Gladysheva I.V., Chertkov K.L., Cherkasov S.V., Khlopko Y.A., Kataev V.Y., Valyshev A.V. (2023). Probiotic potential, safety properties, and antifungal activities of *Corynebacterium amycolatum* ICIS 9 and *Corynebacterium amycolatum* ICIS 53 strains. Probiotics Antimicrob. Proteins.

[44] Yazdi S., Ardekani A.M. (2012). Bacterial aggregation and biofilm formation in a vortical flow. Biomicrofluidics.

[45] Andryukov B.G., Romashko R.V., Efimov T.A., Lyapun I.N., Bynina M.P., Matosova E.V. (2020). Mechanisms of adhesive–cohesive interaction of bacteria in the formation of biofilm. Mol. Genet. Microbiol. Virol..

[46] Idrees M., Sawant S., Karodia N., Rahman A. (2021). *Staphylococcus aureus* biofilm: Morphology, genetics, pathogenesis and treatment strategies. Int. J. Environ. Res. Public Health.

[47] Vetrivel A., Ramasamy M., Vetrivel P., Natchimuthu S., Arunachalam S., Kim G.S., Murugesan R. (2021). *Pseudomonas aeruginosa* biofilm formation and its control. Biologics.

[48] Guerra M.E.S., Destro G., Vieira B., Lima A.S., Ferraz L.F.C., Hakansson A.P., Converso T.R. (2022). *Klebsiella pneumoniae* biofilms and their role in disease pathogenesis. Front. Cell. Infect. Microbiol..

[49] Zomuansangi R., Singh P.K., Singh B.P., Singh G., Deka P., Song J.J., Yadav M.K. (2023). Streptococcus pneumoniae biofilms and human infectious diseases: A review. Understanding Microbial Biofilms.

[50] Cai S., Wu C., Yang W., Liang W., Yu H., Liu L. (2020). Recent advance in surface modification for regulating cell adhesion and behaviors. Nanotechnol. Rev..

[51] Dutta B., Lahiri D., Nag M., Mukherjee D., Ray R.R. (2021). Introduction to bacterial biofilm and acute infections. Biofilm-Mediated Diseases: Causes and Controls.

[52] Bendinger B., Rijnaarts H.H., Altendorf K., Zehnder A.J. (1993). Physicochemical cell surface and adhesive properties of coryneform bacteria related to the presence and chain length of mycolic acids. Appl. Environ. Microbiol..

[53] Burkovski A. (2013). Cell envelope of corynebacteria: Structure and influence on pathogenicity. ISRN Microbiol..

[54] Wang C.C., Mattson D., Wald A. (2001). *Corynebacterium jeikeium* bacteremia in bone marrow transplant patients with Hickman catheters. Bone Marrow Transplant..

[55] Kwaszewska A.K., Brewczynska A., Szewczyk E.M. (2006). Hydrophobicity and biofilm formation of lipophilic skin corynebacteria. Pol. J. Microbiol..

[56] Suzuki T., Iihara H., Uno T., Hara Y., Ohkusu K., Hata H., Ohashi Y. (2007). Suture-related keratitis caused by *Corynebacterium macginleyi*. J. Clin. Microbiol..

[57] Souza M.C., Dos Santos L.S., Sousa L.P., Faria Y.V., Ramos J.N., Sabbadini P.S., Da Santos C.S., Nagao P.E., Vieira V.V., Gomes D.L.R. (2015). Biofilm formation and fibrinogen and fibronectin binding activities by *Corynebacterium pseudodiphtheriticum* invasive strains. Antonie Van Leeuwenhoek.

[58] Merino L., Trejo F.M., De Antoni G., Golowczyc M.A. (2019). Lactobacillus strains inhibit biofilm formation of *Salmonella* sp. isolates from poultry. Food Res. Int..

[59] Zawistowska-Rojek A., Kośmider A., Stępień K., Tyski S. (2022). Adhesion and aggregation properties of Lactobacillaceae strains as protection ways against enteropathogenic bacteria. Arch. Microbiol..

[60] Malfa P., Brambilla L., Giardina S., Masciarelli M., Squarzanti D.F., Carlomagno F., Meloni M. (2023). Evaluation of antimicrobial, antiadhesive and co-aggregation activity of a multi-strain probiotic composition against different urogenital pathogens. Int. J. Mol. Sci..

[61] Borah M., Konwar B.K., Mandal M. (2023). Isolation and characterization of potential probiotic lactic acid bacteria with antimicrobial properties from fermented bamboo shoots of arunachal pradesh. J. Plant Biol. Crop Res..

[62] Idoui T. (2014). Probiotic properties of Lactobacillus strains isolated from gizzard of local poultry. Iran. J. Microbiol..

[63] Hojjati M., Behabahani B.A., Falah F. (2020). Aggregation, adherence, anti-adhesion and antagonistic activity properties relating to surface charge of probiotic *Lactobacillus brevis* gp104 against *Staphylococcus aureus*. Microb. Pathog..

[64] Batoni G., Catelli E., Kaya E., Pompilio A., Bianchi M., Ghelardi E., Di Bonaventura G., Esin S., Maisetta G. (2023). Antibacterial and antibiofilm effects of Lactobacilli strains against clinical isolates of *Pseudomonas aeruginosa* under conditions relevant to cystic fibrosis. Antibiotics.

[65] Paterniti I., Scuderi S.A., Cambria L., Nostro A., Esposito E., Marino A. (2024). Protective effect of probiotics against *Pseudomonas aeruginosa* infection of human corneal epithelial cells. Int. J. Mol. Sci..

[66] Al-Dulaimi M., Algburi A., Abdelhameed A., Mazanko M.S., Rudoy D.V., Ermakov A.M., Chikindas M.L. (2021). Antimicrobial and anti-biofilm activity of polymyxin e alone and in combination with probiotic strains of *Bacillus subtilis* katmira1933 and *Bacillus amyloliquefaciens* b-1895 against clinical isolates of selected *Acinetobacter* spp.: A preliminary study. Pathogens.

[67] Dentice Maidana S., Ortiz Moyano R., Vargas J.M., Fukuyama K., Kurata S., Melnikov V., Villena J. (2022). Respiratory commensal bacteria increase protection against hypermucoviscous carbapenem-resistant *Klebsiella pneumoniae* ST25 infection. Pathogens.

[68] Albarracin L., Ortiz Moyano R., Vargas J.M., Andrade B.G., Cortez Zamar J., Dentice Maidana S., Villena J. (2022). Genomic and immunological characterization of hypermucoviscous carbapenem-resistant *Klebsiella pneumoniae* ST25 isolates from Northwest Argentina. Int. J. Mol. Sci..

[69] Kiryukhina N.V., Melnikov V.G., Suvorov A.V., Morozova Y.A., Ilyin V.K. (2013). Use of *Corynebacterium pseudodiphtheriticum* for elimination of *Staphylococcus aureus* from the nasal cavity in volunteers exposed to abnormal microclimate and altered gaseous environment. Probiotics Antimicrob. Proteins.

[70] Knauf G.A., Powers M.J., Herrera C.M., Trent M.S., Davies B.W. (2022). Acinetobactin-mediated inhibition of commensal bacteria by *Acinetobacter baumannii*. mSphere.

[71] Barzegari A., Kheyrolahzadeh K., Hosseiniyan Khatibi S.M., Sharifi S., Memar M.Y., Zununi Vahed S. (2020). The battle of probiotics and their derivatives against biofilms. Infect. Drug Resist..

[72] Khalfallah G., Gartzen R., Möller M., Heine E., Lütticken R. (2021). A new approach to harness probiotics against common bacterial skin pathogens: Towards living antimicrobials. Probiotics Antimicrob. Proteins.

[73] Yeu J.E., Lee H.G., Park G.Y., Lee J., Kang M.S. (2021). Antimicrobial and antibiofilm activities of *Weissella cibaria* against pathogens of upper respiratory tract infections. Microorganisms.

